# METTL3 Regulates Osteoclast Biological Behaviors via iNOS/NO-Mediated Mitochondrial Dysfunction in Inflammatory Conditions

**DOI:** 10.3390/ijms24021403

**Published:** 2023-01-11

**Authors:** Di Li, Jinlin He, Caihong Fang, Yiwen Zhang, Mingli He, Zhanqi Zhang, Jinsong Hou, Qiong Xu

**Affiliations:** 1 Hospital of Stomatology, Sun Yat-sen University, Guangzhou 510055, China; 2 Guangdong Provincial Key Laboratory of Stomatology, Guangzhou 510055, China; 3 Guanghua School of Stomatology, Sun Yat-sen University, Guangzhou 510055, China

**Keywords:** osteoclast, lipopolysaccharide, m^6^A, mitochondrial function, inducible nitric oxide synthase (iNOS), nitric oxide (NO)

## Abstract

Excessive differentiation of osteoclasts contributes to the disruption of bone homeostasis in inflammatory bone diseases. Methyltransferase-like 3 (METTL3), the core methyltransferase that installs an N6-methyladenosine (m^6^A) modification on RNA, has been reported to participate in bone pathophysiology. However, whether METTL3-mediated m^6^A affects osteoclast differentiation in inflammatory conditions remains unelucidated. In this study, we observed that the total m^6^A content and METTL3 expression decreased during LPS-induced osteoclastogenesis. After knocking down METTL3, we found reduced levels of the number of osteoclasts, osteoclast-related gene expression and bone resorption area. A METTL3 deficiency increased osteoclast apoptosis and pro-apoptotic protein expression. RNA sequencing analysis showed that differentially expressed genes in METTL3-deficient cells were mainly associated with the mitochondrial function. The expression of the mitochondrial function-related genes, ATP production and mitochondrial membrane potential decreased after METTL3 knockdown. Moreover, the most obviously upregulated gene in RNA-Seq was *Nos2*, which encoded the iNOS protein to induce nitric oxide (NO) synthesis. METTL3 knockdown increased the levels of *Nos2* mRNA, iNOS protein and NO content. NOS inhibitor L-NAME rescued the inhibited mitochondrial function and osteoclast formation while suppressing osteoclast apoptosis in METTL3-silenced cells. Mechanistically, a METTL3 deficiency promoted the stability and expression of *Nos2* mRNA, and similar results were observed after m^6^A-binding protein YTHDF1 knockdown. Further in vivo evidence revealed that METTL3 knockdown attenuated the inflammatory osteolysis of the murine calvaria and suppressed osteoclast formation. In conclusion, these data suggested that METTL3 knockdown exacerbated iNOS/NO-mediated mitochondrial dysfunction by promoting a *Nos2* mRNA stability in a YTHDF1-dependent manner and further inhibited osteoclast differentiation and increased osteoclast apoptosis in inflammatory conditions.

## 1. Introduction

Skeletal integrity and function rely on bone homeostasis, which is sustained by coordinating the dynamic balance between osteoclastic bone degradation and osteoblastic bone deposition [1]. Osteoclasts originate from the hematopoietic monocyte/macrophage lineage, which are multinucleated cells possessing the unique capacity for bone resorption [2]. In inflammatory conditions, osteoclastic bone resorption is overactivated by pathogens and virulence factors, which contributes to the development of pathological bone disorders such as rheumatoid arthritis, osteomyelitis and periodontitis [3]. Lipopolysaccharide (LPS), a characteristic component of Gram-negative bacteria, has been reported to be a potent inducer of inflammatory osteolysis. LPS can recruit host immune cells for the generation of various inflammatory cytokines and promote osteoclast differentiation, activation and survival, leading to bone destruction in infectious bone diseases [4].

N6-metyladenosine (m^6^A), the methylation of nitrogen-6 positions in adenosine, is a widespread modification of mRNA in most eukaryotes [5]. Research based on m^6^A-specific methylated immunoprecipitation and high-throughput sequencing has identified the consensus motif RRACH ([G/A/U] [G > A] AC [U > A > C]) as the core sequence content of the m^6^A site [6,7]. As a dynamically reversible modification, m^6^A is installed by a methyltransferase complex composed of the core enzyme methyltransferase-like 3 (METTL3) and accessory subunits, while it is removed by demethylases. YTH structural domain binding proteins (YTHDFs) can recognize and interact with the m^6^A-modified mRNA to influence mRNA processing, such as nuclear export, degradation and translation [8]. A growing number of studies have demonstrated the importance of m^6^A modification in cellular biological processes, including the macrophage immune response, hematopoietic stem cell differentiation and myeloid leukemia cell apoptosis [9,10,11]. Recent studies have unveiled that METTL3-mediated m^6^A modification is linked to inflammatory bone diseases, but the role of METTL3 can vary in different cells and tissues. It has been reported that METTL3 expression is elevated in peripheral blood mononuclear cells from rheumatoid arthritis (RA) patients, as well as in LPS-stimulated macrophage-like cells (pTHP-1). The overexpression of METTL3 attenuates the inflammatory response induced by LPS in pTHP-1 macrophages [12]. The expression of METTL3 is also upregulated in the synovial tissues from RA patients and in fibroblast-like synoviocytes (FLSs) treated with TNF-α. However, METTL3 acts as a facilitator of an inflammatory response in FLSs [13], which seems inconsistent with the role of METTL3 in pTHP-1 cells. More interestingly, a study by Sang et al. reported that METTL3 expression was reduced in knee tissues from osteoarthritis (OA) patients and in inflammatory cytokine-stimulated chondrocytes. METTL3 overexpression reduced the levels of inflammatory cytokines in the chondrosarcoma cell line SW1353 [14]. These results suggested that the role of METTL3 in inflammatory environments may exhibit tissue and cell-type specificity. As the main cells that perform the function of bone resorption in inflammatory osteolytic diseases, whether the biological behavior of osteoclasts is modulated by METTL3 under inflammatory conditions has not been elucidated.

Mitochondria are membrane-enclosed organelles that generate the majority of cellular energy in the form of adenosine triphosphate (ATP). In addition to energy production, mitochondria participate in various biological functions, including cellular differentiation, inflammatory responses, redox equilibrium and endogenous apoptosis [15,16]. As highly differentiated multinuclear cells, osteoclasts are unique in their ability to degrade bone mineral and collagen matrices by secreting lytic enzymes, and the process of osteoclast bone resorption consumes a substantial quantity of energy. Accordingly, a high abundance of mitochondria can be observed in osteoclasts and the appropriate mitochondrial function is critical for effective osteoclast differentiation and activity [17,18,19]. Rotenone, a mitochondrial respiratory chain inhibitor, was found to reduce the number of osteoclasts and the bone resorption lacunae area in vitro, and inhibit murine bone loss induced by LPS in vivo [20]. Mitochondrial dysfunction caused by the depletion of the essential mitochondrial complex subunits Ndufs4 confers resistance to inflammatory bone erosion via an impairment of osteoclastogenesis and bone resorption [21]. As one of the most susceptible organelles during inflammation-mediated cell dysfunction [22], whether mitochondria participate in the process of m^6^A-regulated inflammatory osteoclastogenesis remains unknown.

In this study, we utilized LPS to construct an inflammatory osteoclastogenesis model in vitro and an osteolysis animal model in vivo, with a focus on exploring the regulatory effects of METTL3 on inflammatory osteoclastogenesis and the role of mitochondrial function in this process.

## 2. Results

### 2.1. METTL3 Expression Is Downregulated during Inflammatory Osteoclastogenesis

**Figure 1:**
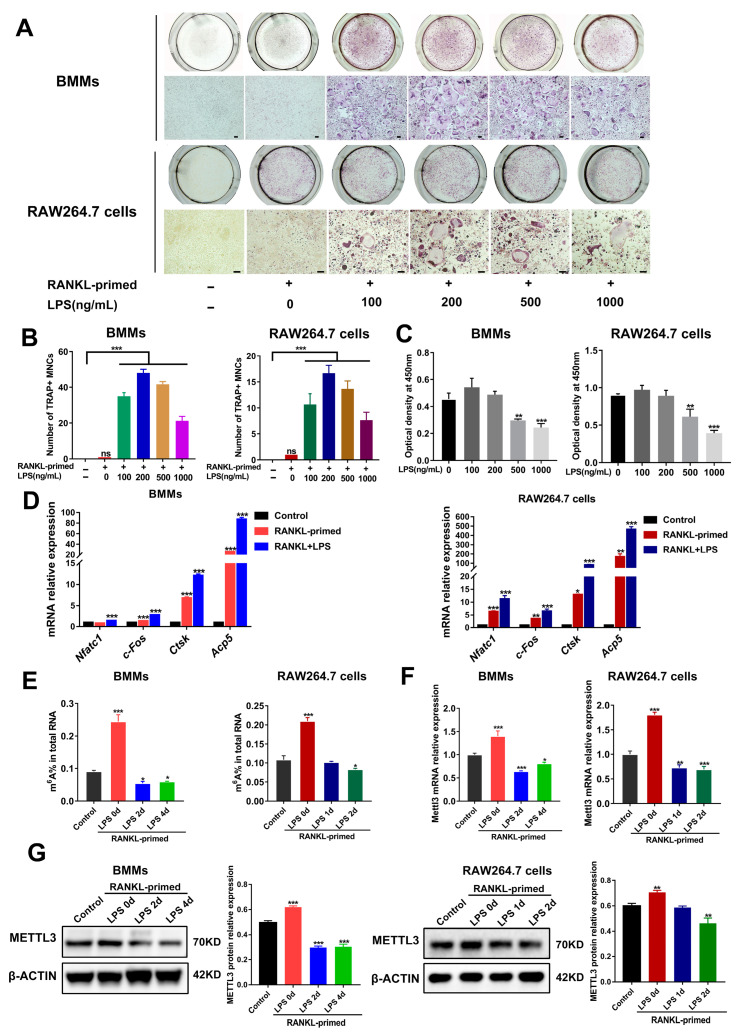
METTL3 expression during inflammatory osteoclastogenesis induced by LPS. (**A**) BMMs and RAW264.7 cells were pretreated with 50 ng/mL RANKL and then stimulated with 0–1000 ng/mL LPS. TRAP staining was performed to visualize osteoclasts. (**B**) Multinucleated cells with TRAP-positive were considered mature osteoclasts. Scale bars, 100 μm. MNCs, multinucleated cells. (**C**) Cell viability was tested with a CCK8 assay after cells were treated with the indicated concentrations of LPS for 4 days. (**D**–**F**) RANKL-primed osteoclast precursors were stimulated with 200 ng/mL LPS for 0–4 days. Cells treated without RANKL and LPS acted as the control. (**D**) qRT-PCR was performed to detect the mRNA expression of *Nfatc1*, *c-Fos*, *Ctsk* and *Acp5*. (**E**) Total m^6^A levels were measured using m^6^A RNA methylation quantification. (**F**) qRT-PCR was performed to measure Mettl3 mRNA expression in BMMs and RAW264.7 cells stimulated by LPS for the indicated time. (**G**) The METTL3 protein levels during LPS-induced osteoclastogenesis were assessed with Western blotting. β-Actin was used as an internal control for qRT-PCR and Western blotting. The data represent the mean ± SD of three independent experiments (*n* = 3). ns, no significance; * *p* < 0.05; ** *p <* 0.01; *** *p* < 0.001.

To determine whether METTL3 participates in osteoclast development modulation under inflammatory environments, we first constructed an in vitro model of LPS-induced osteoclastogenesis. As determined using TRAP staining, after bone marrow-derived macrophages (BMMs) and RAW264.7 cells were pretreated with RANKL (50 ng/mL), the stimulation of LPS (100–1000 ng/mL) increased the number of TRAP-positive multinucleated cells (Figure 1A,B). LPS at a 200 ng/mL concentration had no effect on the cell viability of osteoclast precursors, as suggested using a CCK8 assay (Figure 1C). Accordingly, we chose 200 ng/mL LPS to induce inflammatory osteoclastogenesis in subsequent experiments. The data from qRT-PCR showed that the mRNA expression of osteoclastic transcription factors *Nfatc1* and *c-Fos*, as well as osteoclastic markers *Acp5* and *Ctsk*, was promoted through LPS stimulation in RANKL-pretreated cells (Figure 1D). Thus, we confirmed that LPS promoted osteoclast formation in RANKL-pretreated osteoclast precursors and successfully established an in vitro inflammatory osteoclastogenesis model.

We next analyzed the levels of m^6^A and METTL3 during inflammatory osteoclast differentiation. The data suggested that, in comparison with the control group, the m^6^A content increased in RANKL-pretreated cells but decreased upon LPS stimulation (Figure 1E). Consistent with this, LPS-treated cells showed a significantly decreased expression of METTL3 mRNA and protein (Figure 1F,G), implying a putative role for METTL3 in modulating osteoclastogenesis under inflammatory conditions.

### 2.2. METTL3 Knockdown Regulates Osteoclast Differentiation, Bone Resorption Capacity and Apoptosis under Inflammatory Conditions

**Figure 2:**
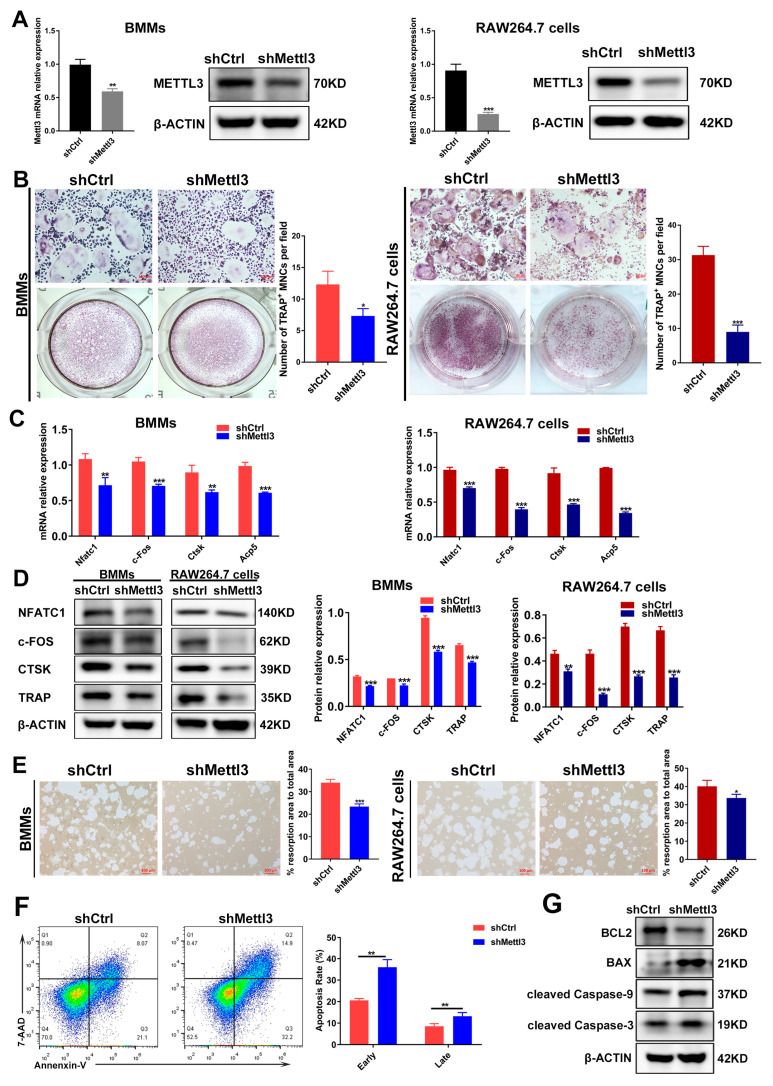
Effect of METTL3 knockdown on osteoclast differentiation, bone resorption capacity and osteoclast apoptosis under inflammatory conditions. (**A**) qRT-PCR and Western blotting were utilized to assess the efficiency of METTL3 knockdown. shCtrl, cells transfected with the negative control shRNA; shMettl3, cells transfected with the shRNA targeting *Mettl3*. (**B**–**G**) BMMs and RAW264.7 cells with or without METTL3 knockdown were induced to differentiate into osteoclasts as described previously. (**B**) Images of TRAP staining. Scale bars, 100 μm. (**C**) The mRNA expression of *Nfatc1*, *c-Fos*, *Ctsk* and *Acp5* was assessed by qRT-PCR. (**D**) The protein levels of NFATC1, c-FOS, CTSK and TRAP were detected using Western blotting. (**E**) Osteoclast bone-resorbing capacity was assessed using pit formation assay. Quantification of the resorption area is shown. Scale bars, 100 μm. (**F**) Apoptosis of osteoclasts differentiated from RAW264.7 cells was detected using flow cytometry analysis. Early apoptotic cells were stained Annexin V-positive and 7AAD-negative, while late apoptotic cells were Annexin V-positive and 7AAD-positive. (**G**) Western blotting was performed to evaluate the protein levels of BCL2, BAX and cleaved caspase-9, -3. β-Actin was used as an internal control for qRT-PCR and Western blotting. The data are shown as the mean ± SD (*n* = 3). * *p* < 0.05; ** *p* < 0.01; *** *p* < 0.001.

It has been demonstrated that the role of METTL3 is inconsistent in different cells and tissues [23]. The experimental data from our previous study also evidenced the differential role of METTL3 in human dental pulp cells (HDPCs) and mouse macrophages [24,25]. In LPS-treated HDPCs, the expression of METTL3 was elevated, and METTL3 knockdown reduced the LPS-induced expression of inflammatory cytokines by increasing the expression of MyD88S, a splicing variant of MyD88 that inhibits inflammatory cytokine production [24]. Conversely, METTL3 expression levels decreased in macrophages stimulated with LPS, and METTL3 knockdown promoted inflammatory cytokine expression by enhancing mRNA stability and the expression of NOD1 and RIPK2, which, consequently, upregulated NOD1 signaling [25]. To further explore the potential role of METTL3 in inflammatory osteoclastogenesis, short hairpin RNA (shRNA) was used to inhibit METTL3 expression in BMMs and RAW264.7 cells. The expression of METTL3 mRNA and protein markedly decreased in the shMettl3 group (Figure 2A), confirming the efficiency of METTL3 knockdown. After a METTL3 deficiency was reached, BMMs and RAW264.7 cells were pretreated with RANKL and stimulated with LPS to be differentiated into osteoclasts. As shown by TRAP staining, the knockdown of METTL3 reduced the number of mature osteoclasts (Figure 2B). The mRNA and protein levels of osteoclast-related genes, including Nfatc1, c-Fos, Acp5 and Ctsk, also decreased in the shMettl3 group compared with the shRNA control group (Figure 2C,D). A pit formation assay was performed to assess the effect of METTL3 on LPS-induced osteoclast function. The results displayed a significant reduction in the bone resorption area after METTL3 knockdown (Figure 2E). In addition, the flow cytometry and quantification analysis showed that the early and late cell apoptosis rate increased in the shMettl3 group (Figure 2F). Similarly, Figure 2G shows that the anti-apoptotic protein BCL2 level decreased, while levels of pro-apoptotic protein BAX and apoptotic markers cleaved caspase-9 and cleaved caspase-3 increased in *Mettl3*-deficient cells, further confirming the aggravated cell apoptosis caused by METTL3 knockdown. These data indicated that METTL3 knockdown impeded osteoclast differentiation as well as the bone-resorbing activity, and promoted osteoclast apoptosis under inflammatory conditions.

### 2.3. METTL3 Deficiency Triggers Mitochondrial Dysfunction during LPS-Induced Osteoclastogenesis

**Figure 3:**
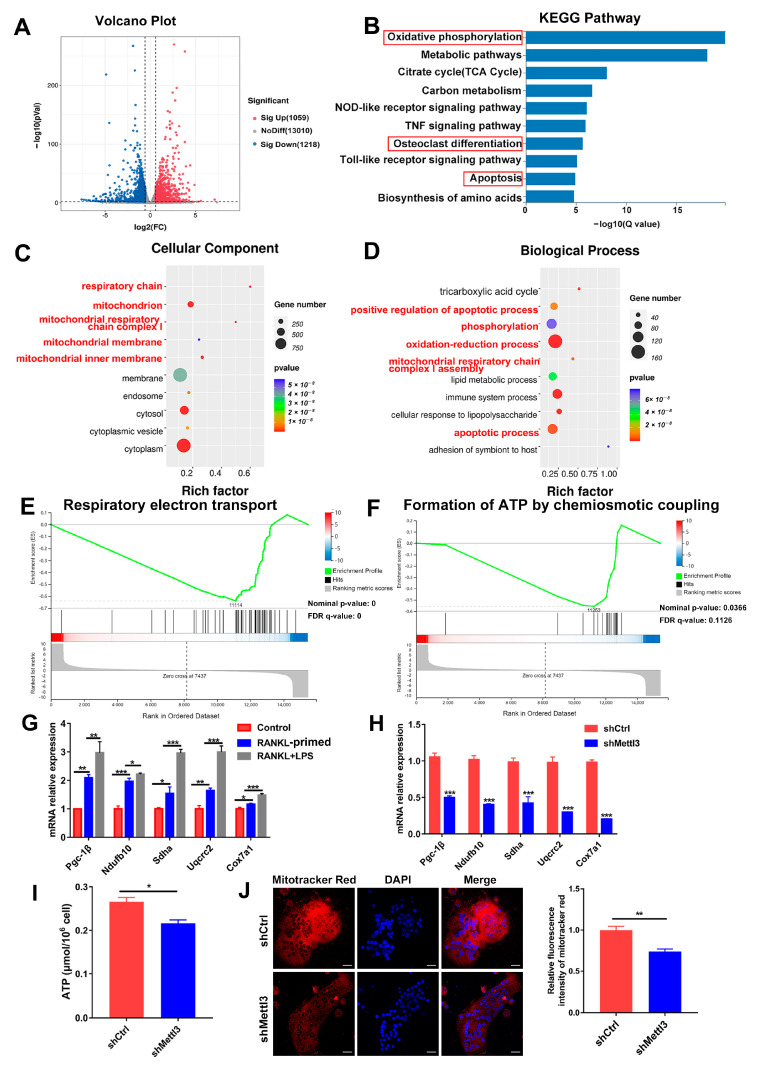
Effect of METTL3 deficiency on mitochondrial homeostasis during LPS-induced osteoclastogenesis. (**A**) The volcano plot exhibited differentially expressed genes (DEGs) between the shMettl3 and shCtrl groups (*p* < 0.01, fold change >1.5 or <0.67, compared to the shCtrl group). (**B**) Enriched KEGG pathways of DEGs. (**C**,**D**) GO analysis bubble charts of cellular components and biological processes showing the top ten enriched terms of DEGs. (**E**,**F**) The GSEA enrichment plots of mitochondrial function-related gene sets, including respiratory electron transport (**E**) and formation of ATP through chemiosmotic coupling (**F**). The nominal *p*-value and false discovery rate (FDR) are shown in plots. Significance threshold was set to *p*-value < 0.05, FDR < 0.25. (**G**) qRT-PCR was performed to detect the mRNA expression of *Pgc-1β*, *Ndufb10*, *Sdha*, *Uqcrc2* and *Cox7a1* during LPS-induced osteoclastogenesis. (**H**–**J**) Osteoclast precursors transfected with the *Mettl3* shRNA or negative control shRNA were induced to differentiate into osteoclasts. (**H**) *Pgc-1β*, *Ndufb10*, *Sdha*, *Uqcrc2* and *Cox7a1* mRNA levels were measured using qRT-PCR. *β-Actin* was used as an internal normalized control. (**I**) ATP content was detected using an ATP assay kit. (**J**) Confocal images of osteoclasts stained with Mito-Tracker Red CMXRos and DAPI. Scale bars, 25 μm. The relative fluorescence intensity of Mito-Tracker Red was analyzed with the ImageJ software. The results are shown as the mean ± SD (*n* = 3). * *p* < 0.05; ** *p* < 0.01; *** *p* < 0.001.

To elucidate the regulatory mechanism of METTL3 in osteoclast biological behavior, RNA sequencing and bioinformatics analysis were performed in shRNA-transfected cells after LPS-induced osteoclastogenesis. A total of 2277 differentially expressed genes (DEGs) between the METTL3-deficient group and control group were identified, among which 1059 were upregulated and 1218 were downregulated (Figure 3A). KEGG analysis revealed that the DEGs were enriched in the pathways of oxidative phosphorylation, osteoclast differentiation and apoptosis (Figure 3B). The GO analysis showed that METTL3-regulated DEGs encoded proteins localized in the mitochondrion and involved in the processes of oxidation–reduction, phosphorylation, and mitochondrial respiratory chain complex assembly (Figure 3C,D). The GSEA data displayed that the mitochondrial function, including respiratory electron transport and ATP formation, was inhibited in the METTL3-knockdown group (Figure 3E,F). These results of the bioinformatic analysis suggested that METTL3 influenced mitochondrial function in LPS-induced osteoclasts.

It Is well-known that mitochondrial energy metabolism produces abundant ATP to support cell proliferation, differentiation and activity. Dysfunction of the mitochondria impairs energy generation and causes endogenous cell apoptosis [26]. Expression of the mitochondrial energy metabolism transcription factor *Pgc-1β*, as well as mitochondrial respiratory chain complex genes *Ndufb10*, *Sdha*, *Uqcrc2* and *Cox7a1*, was upregulated in osteoclasts upon LPS treatment, confirming the essential role of mitochondrial function for inflammatory osteoclastogenesis (Figure 3G). Next, we explored whether METTL3 affects mitochondrial function in LPS-induced osteoclasts. As assessed using qRT-PCR, the mRNA expression of *Pgc-1β*, *Ndufb10*, *Sdha*, *Uqcrc2* and *Cox7a1* was reduced after METTL3 knockdown (Figure 3H). The mitochondrial activity of ATP production was also significantly impaired in the shMettl3 group (Figure 3I). LPS-induced osteoclasts were incubated with Mito-Tracker Red CMXRos, which labeled mitochondria in a manner dependent on the mitochondrial membrane potential. The results showed that red fluorescence intensity markedly decreased in METTL3-knockdown cells (Figure 3J), indicating the decreased mitochondrial membrane potential. Taken together, these data demonstrated that METTL3 ablation in LPS-treated osteoclasts led to mitochondrial dysfunction.

### 2.4. Mitochondrial Dysfunction in METTL3-Deficient Osteoclasts Is Mediated by iNOS/NO Signaling

**Figure 4:**
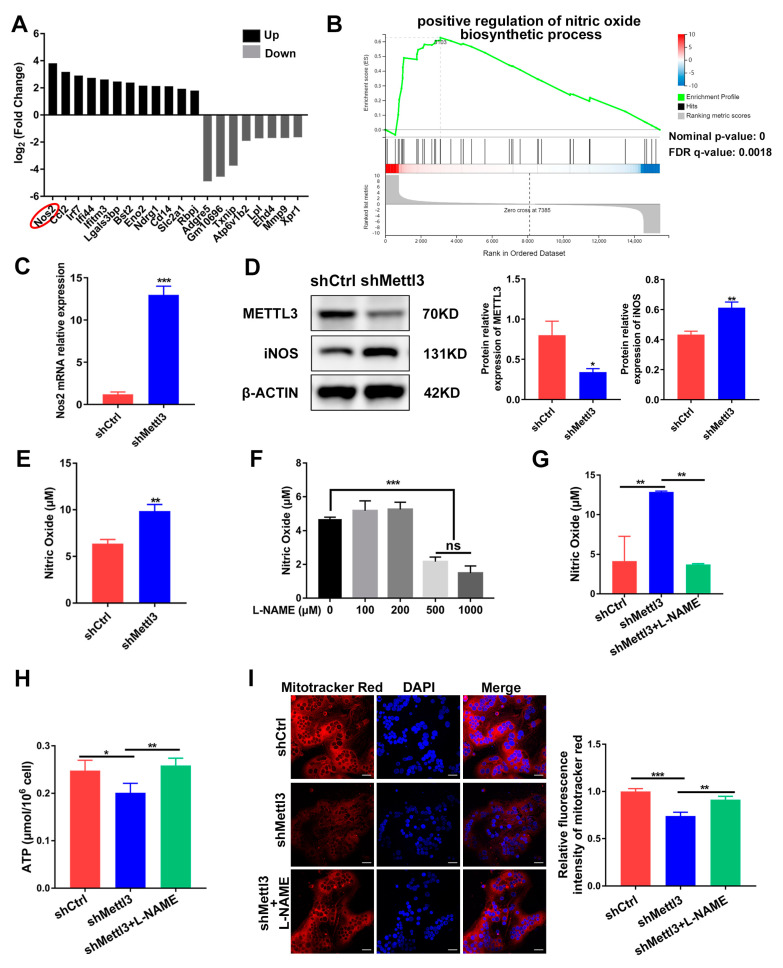
Identification of iNOS/NO signaling as a mediator of mitochondrial dysfunction in METTL3-deficient cells. (**A**) The top 20 differentially expressed genes from RNA sequencing data between the shMettl3 group and the shCtrl group. (**B**) The GSEA enrichment plot of the gene sets associated with positive regulation of nitric oxide biosynthetic process. (**C**) *Nos2* mRNA expression was assessed using qRT-PCR. (**D**) iNOS protein level was determined with Western blotting. β-Actin was used as an internal control. (**E**) The Griess assay was performed to detect the production of nitric oxide. (**F**) RAW264.7 cells were treated with LPS (200 ng/mL) and different concentrations of L-NAME (0–1000 μM) for 2 days, and the NO content was assessed through the Griess assay. (**G**–**I**) RANKL-pretreated osteoclast precursors transfected with the indicated shRNA were treated with LPS (200 ng/mL) in the absence or presence of L-NAME (500 μM) for 2 days. (**G**) The NO content was analyzed using the Griess assay. (**H**) ATP concentration was measured using an ATP assay kit. (**I**) Confocal images of cells stained with Mito-Tracker Red CMXRos and DAPI. Scale bars, 25μm. The relative fluorescence intensity of Mito-Tracker Red was analyzed using the ImageJ software. The data are shown as the mean ± SD (*n* = 3). ns, no significance; * *p* < 0.05; ** *p* < 0.01; *** *p* < 0.001.

To further clarify the potential downstream target of METTL3 during inflammatory osteoclastogenesis, we sorted the DEGs screened using RNA sequencing according to their fold change. The top 20 DEGs were depicted in Figure 4A, with *Nos2* being the most significantly upregulated. *Nos2* encodes the inducible nitric oxide synthase (iNOS) to catalyze nitric oxide (NO) production, which has been reported to act as a negative regulator of mitochondrial function integrity and participate in cell fate modulation [27]. GSEA analysis of RNA-Seq showed a significant promotion of the nitric oxide biosynthetic process in the shMettl3 group (Figure 4B). As verified using qRT-PCR and Western blotting, *Nos2* mRNA and iNOS protein levels in the shMettl3 group were elevated (Figure 4C,D). Moreover, a notable increase in NO content was observed following the upregulation of iNOS expression caused by METTL3 knockdown (Figure 4E).

To explore the biological contribution of iNOS/NO signaling in METTL3-regulated mitochondrial homeostasis in an inflammatory environment, LPS-stimulated osteoclast precursors were treated with the NOS inhibitor L-NAME to prevent NO production. The amount of NO was measured after cells were treated with 0–1000 μM L-NAME. A significant reduction of NO content was observed at a minimum concentration of 500 μM, which was utilized in further experiments (Figure 4F). The elevated level of NO caused by METTL3 knockdown was abolished upon L-NAME treatment, confirming its inhibitory effect (Figure 4G). L-NAME rescued the inhibited ATP production caused by METTL3 knockdown in LPS-treated osteoclasts (Figure 4H). As determined using the fluorescence intensity of Mito-Tracker Red in osteoclasts, METTL3 knockdown resulted in reduced mitochondrial membrane potential, which was enhanced by L-NAME treatment (Figure 4I). Overall, these results suggested that iNOS/NO signaling mediated mitochondrial homeostasis disruption in METTL3-knockdown cells, which may be involved in the mechanisms through which METTL3 influences inflammatory osteoclast development.

### 2.5. Inhibiting iNOS/NO Signaling Enhances Osteoclast Differentiation and Decreases Apoptosis in METTL3-Knockdown Cells

**Figure 5:**
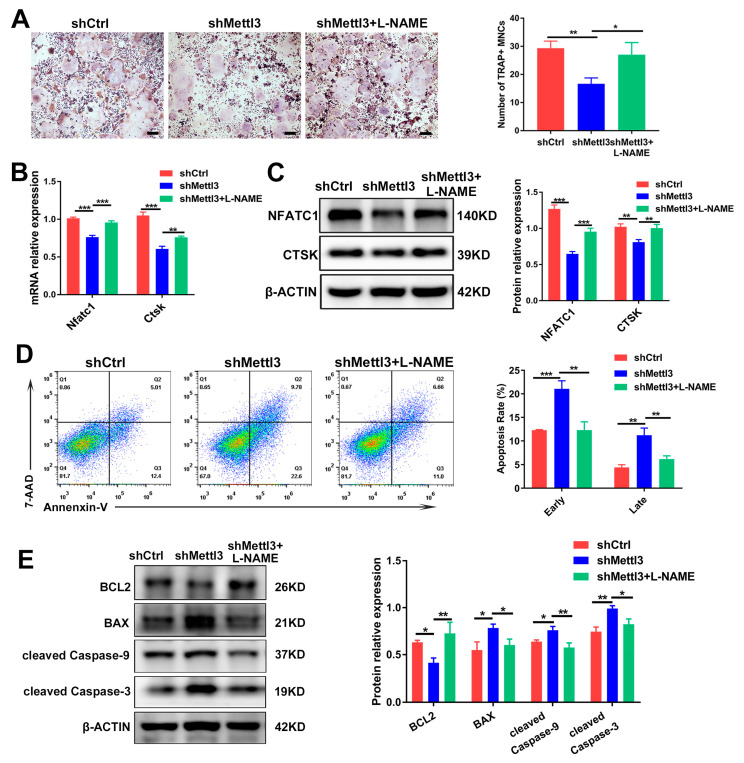
The rescuing effect of L-NAME on osteoclast differentiation and apoptosis in METTL3-silenced cells treated with LPS. Osteoclast precursors transfected with shRNA and pretreated with RANKL were differentiated into osteoclasts by LPS (200 ng/mL) induction in the absence or presence of L-NAME (500 μM). (**A**) Images of TRAP staining. Scale bars, 200 μm. The mRNA and protein levels of NFATC1 and CTSK were assessed using qRT-PCR (**B**) and Western blotting (**C**). (**D**) The apoptosis rate of osteoclasts was determined by flow cytometry analysis. (**E**) The protein levels of BCL2, BAX, cleaved caspase-9 and cleaved caspase-3 were evaluated using Western blotting. β-Actin was used as an internal control for qRT-PCR and Western blotting. The data are shown as the mean ± SD (*n* = 3). * *p* < 0.05; ** *p* < 0.01; *** *p* < 0.001.

To demonstrate whether METTL3 modulates inflammatory osteoclast differentiation and apoptosis through iNOS/NO signaling, we stimulated LPS-induced cells with L-NAME, which inhibited iNOS activity and NO generation. As determined using TRAP staining, L-NAME rescued the reduction of the mature osteoclast number in METTL3-deficient cells (Figure 5A). After impeding NO generation in METTL3-knockdown cells, the expression of the osteoclast differentiation markers NFATC1 and CTSK indicated an improved osteoclastogenesis potential (Figure 5B,C). Meanwhile, the flow cytometry analysis revealed that the upregulated cell apoptosis rate in the shMettl3 group was reversed by L-NAME (Figure 5D). Mitochondria-related apoptotic protein expression was examined using Western blotting, and the results showed that pro-survival protein BCL2 expression increased, while the expression of pro-apoptotic protein BAX and apoptotic markers cleaved caspase-9 and cleaved caspase-3 decreased upon L-NAME treatment in the shMettl3 group (Figure 5E). Together, these findings indicated that METTL3 knockdown attenuated osteoclast formation and enhanced cell apoptosis during inflammatory osteoclastogenesis, which might be due to the promoted iNOS/NO signaling-mediated mitochondrial dysfunction.

### 2.6. METTL3 Induces Nos2 mRNA Degradation in a YTHDF1-Dependent Manner

**Figure 6:**
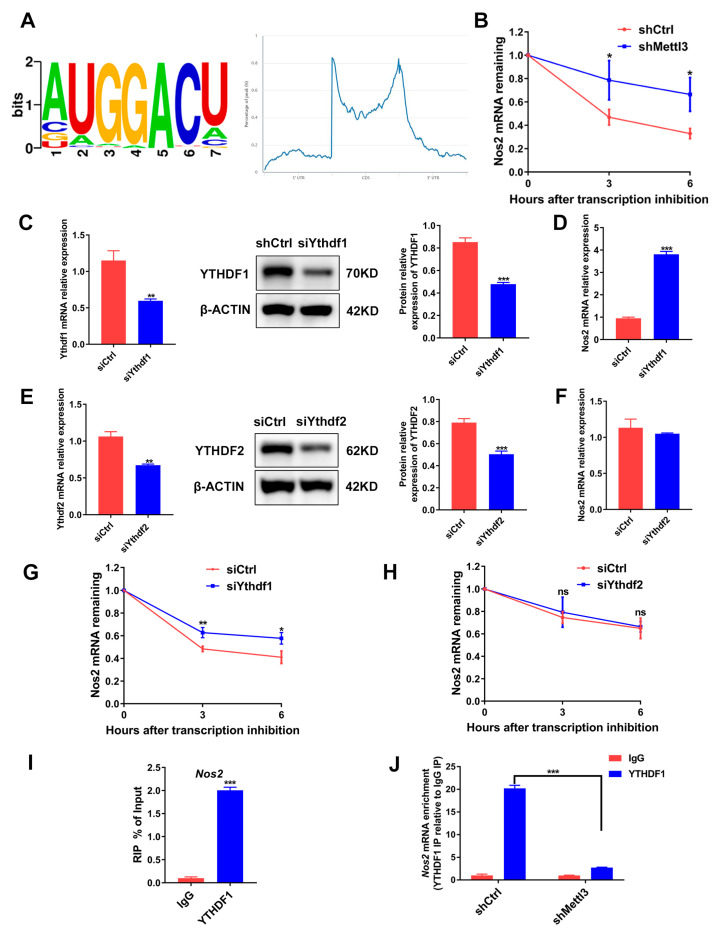
The role of METTL3, YTHDF1 and YTHDF2 in the degradation of *Nos2* mRNA. (**A**) RMBase 2.0 database analysis of m^6^A motifs and m^6^A peak distribution on *Nos2* transcript. (**B**) RAW264.7 cells transfected with shRNA were differentiated into osteoclasts. Then, cells were stimulated with actinomycin D (5 μg/mL) to inhibit transcription. The *Nos2* mRNA levels at the indicated time after actinomycin D treatment were assessed using qRT-PCR. (C–H) RAW264.7 cells transfected with siRNA were induced to differentiate into osteoclasts. (**C**,**E**) YTHDF1 and YTHDF2 protein levels were determined with Western blotting after siRNA-mediated gene knockdown. (**D**,**F**) *Nos2* mRNA expression in the si*Ythdf1* group or the si*Ythdf2* group was measured with qRT-PCR. (**G**,**H**) Osteoclast precursors were treated with actinomycin D (5 μg/mL) for the indicated time following inflammatory osteoclastogenesis. The mRNA expression of *Nos2* was determined using qRT-PCR. Cells transfected with the negative control siRNA acted as the control group. (**I**,**J**) A RIP-qPCR assay using an IgG antibody and an anti-YTHDF1 antibody was performed to determine the binding of *Nos2* mRNA with YTHDF1. β-Actin was used as an internal control for qRT-PCR and Western blotting. The data are shown as the mean ± SD (*n* = 3). ns, no significance; * *p* < 0.05; ** *p* < 0.01; *** *p* < 0.001.

METTL3-dependent m^6^A methylation on mRNAs participates in RNA processing to regulate mRNA splicing, localization, stability and translation [28]. Through querying the online bioinformatics tool RMBase 2.0 database (https://rna.sysu.edu.cn/rmbase/, accessed on 15 February 2021) we identified the consensus motifs for m^6^A modification on *Nos2* mRNA, suggesting that *Nos2* mRNA might be the target gene modified by METTL3 (Figure 6A). As *Nos2* and METTL3 expression was negatively correlated, we hypothesized that METTL3 might decrease *Nos2* mRNA stability. Hence, we analyzed *Nos2* mRNA expression in cells treated with the transcription inhibitor actinomycin D. Compared with the shCtrl group, the *Nos2* mRNA level was significantly higher in the shMettl3 group (Figure 6B), confirming that METTL3 knockdown stabilized *Nos2* mRNA.

m^6^A readers YTHDF1 and YTHDF2 were identified to recognize and affect the stability of the m^6^A-modified mRNA [29]. To determine whether YTHDF1 or YTHDF2 regulates *Nos2* expression through mRNA degradation, we knocked down YTHDF1 or YTHDF2 in osteoclast precursor cells (Figure 6C,E). Similar to METTL3 silencing, YTHDF1 knockdown markedly elevated *Nos2* expression (Figure 6D), while YTHDF2 knockdown did not affect the *Nos2* mRNA level (Figure 6F). The mRNA stability assays further demonstrated that YTHDF1 knockdown, instead of YTHDF2 knockdown, increased mRNA stability (Figure 6G,H). RIP-qPCR was performed to confirm whether YTHDF1 directly interacts with *Nos2*, and the results showed that *Nos2* mRNA was remarkedly enriched in the anti-YTHDF1 group (Figure 6I). METTL3 knockdown decreased the *Nos2* mRNA enrichment, indicating that the binding of YTHDF1 with *Nos2* was dependent on METTL3-mediated m^6^A methylation (Figure 6J). In summary, the data suggested that an METTL3 deficiency enhanced iNOS/NO signaling through the upregulation of *Nos2* mRNA stability and expression in a YTHDF1-dependent manner.

### 2.7. METTL3 Lentiviral Interference Suppresses Bone Destruction in an LPS-Induced Calvaria Osteolysis Murine Model

**Figure 7:**
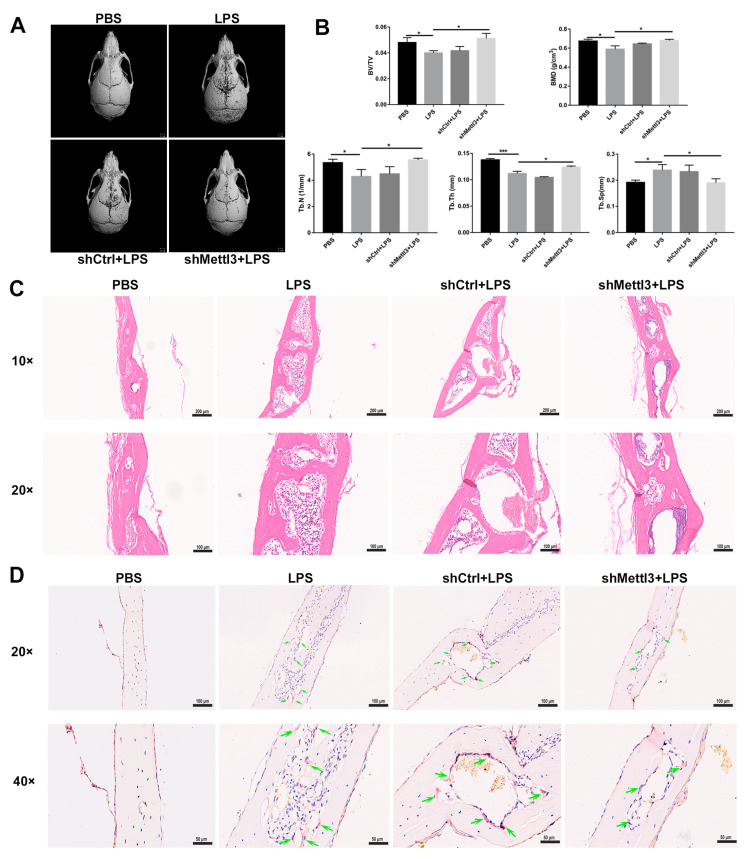
The effect of METTL3 interference on LPS-induced osteolysis in mouse calvaria. (**A**) Representative micro-CT reconstruction images of the murine calvaria from each group. (**B**) Quantitative analysis of the bone volume against tissue volume (BV/TV), bone mineral density (BMD), trabecular number (Tb. N), trabecular thickness (Tb. Th) and trabecular separation (Tb. Sp). (**C**,**D**) The calvarial bone slices were stained with H&E and TRAP. (**C**) Histological slide images of H&E staining at 10× and 20× magnification. (**D**) Images of TRAP staining at 20× and 40× magnification. Upper scale bars, 100 μm; lower scale bars, 50 μm. The green arrows indicate osteoclasts. The data represent the mean ± SD (*n* = 3). * *p* < 0.05; *** *p* < 0.001.

To evaluate the potential effect of METTL3 on pathological bone loss in vivo, a well-characterized murine model of LPS-induced calvaria osteolysis was established as described previously [30]. The reconstruction images of Micro-CT scanning showed extensive bone destruction on the calvaria surface in the LPS group compared with the PBS group (Figure 7A). A quantitative analysis of bone morphometry further confirmed that BV/TV, BMD, Tb. N and Tb. Th decreased, while Tb. Sp increased in the LPS-treated group (Figure 7B). In contrast, a local injection of lentivirus carrying *Mettl3*-shRNA markedly inhibited LPS-induced osteolytic defects (Figure 7A). The decrease in BV/TV, BMD, Tb. N and Tb. Th, and the increase in Tb. Sp caused by the LPS injection were also restored through *Mettl3*-shRNA lentivirus administration (Figure 7B). Consistent with the results from Micro-CT, H&E staining showed that obvious bone erosion and inflammatory cells infiltration were observed in the LPS-injected group, whereas calvaria bone destruction and inflammation were alleviated after *Mettl3*-shRNA interference (Figure 7C). TRAP staining revealed that abundant TRAP-positive cells presented on the eroded bone surface in the LPS group, while an injection of *Mettl3*-shRNA lentivirus decreased the formation of TRAP-positive cells (Figure 7D). Together, these results indicated that an injection of lentivirus-mediated shRNA targeting *Mettl3* attenuated local inflammatory osteolysis, at least partly via the inhibition of osteoclast formation.

## 3. Discussion

Inflammatory bone diseases, such as osteomyelitis and periodontitis, are characterized by increased osteoclast formation and potent bone erosion, which compromise the skeleton’s stability and impose a health concern [31]. LPS has been recognized as the predominant virulent factor that triggers the activation of osteoclasts and causes infective osteolysis in vivo [4]. Studies have illustrated that once osteoclast precursors were committed to the osteoclast lineage by RANKL, LPS could promote osteoclast formation in vitro [32,33]. In the present study, we established an in vitro model of inflammatory osteoclastogenesis by stimulating RANKL-pretreated osteoclast precursors with LPS. The results showed that LPS accelerated the osteoclastogenesis of RANKL-primed cells, characterized by the increased number of TRAP-positive multinucleated cells and the elevated expression of osteoclastic genes.

M^6^A occurring at the N6 position of adenosine is a widespread methylation modification in eukaryotic mRNA, which has recently become a major research hotspot of epigenetics due to its pivotal function in physiological processes and disease progression [34]. METTL3, which was the first identified component of the m^6^A methyltransferase complex (MTC), binds to the methyl donor and acts as the catalytic core to modulate m^6^A modification [35]. Recent studies suggested that METTL3-modified m^6^A contributes to bone homeostasis by modulating gene expression in bone-related cells [36,37]. Although METTL3-modified m^6^A has been widely identified as participating in osteoblast differentiation, its role in osteoclast development has received little attention. Our previous studies showed that the expression of METTL3 increased during osteoclast differentiation; the knockdown of METTL3 inhibited RANKL-induced osteoclast formation through mechanisms involving *Traf6* mRNA nuclear export and YTHDF2-mediated mRNA degradation in physiological conditions [38]. To further illustrate whether METTL3-mediated m^6^A is involved in modulating osteoclast biological behaviors in inflammatory environments, we analyzed the expression pattern of m^6^A and METTL3 during osteoclastogenesis with LPS stimulation. The results showed that the overall levels of m^6^A and METTL3 increased upon RANKL pretreatment and decreased after LPS stimulation during inflammatory osteoclastogenesis. To explore the regulatory effect of METTL3 on osteoclast biological behaviors, we knocked down METTL3 expression in osteoclast precursors. The results indicated that METTL3 silencing decreased the number of osteoclasts and bone resorption area. The expression of osteoclast-related genes, such as Nfatc1, c-Fos, Ctsk and Acp5, also decreased after METTL3 knockdown. Among these osteoclastic molecules, NFATc1 serves as a master transcription factor required for osteoclastogenesis by activating the expression of osteoclast-specific genes, leading to the differentiation of mononuclear osteoclast precursors into TRAP positive multinuclear cells [39,40]. The reduced expression of this key transcription factor of osteoclastogenesis offers a possible explanation for the inhibited osteoclast differentiation caused by METTL3 knockdown. In addition, the osteoclast apoptosis rate and levels of pro-apoptotic proteins increased while the expression of anti-apoptotic proteins decreased after METTL3 silencing. These data suggested that METTL3 knockdown suppressed osteoclast differentiation and the bone resorption function, and accelerated osteoclast apoptosis under the LPS-stimulated inflammatory conditions.

To decipher the underlying mechanisms by which METTL3 modulates osteoclast biological behaviors in the inflammatory microenvironment, we performed RNA sequencing in osteoclasts with and without METTL3 knockdown. The bioinformatics analysis revealed that the regulatory effect of METTL3 on the biological behavior of LPS-treated osteoclasts may be attributed to a mitochondrial dysfunction. Mitochondria are double-membrane organelles known as “energy generators” of the cells, converting nutrients and oxygen into ATP through oxidative phosphorylation [41]. As the master intracellular power producer, the proper function of mitochondria is critical for biological processes demanding energy, including cell survival, differentiation and activation [42,43]. Osteoclast differentiation is a process with high energy demand, which is supported by the substantial amount of ATP generated by mitochondrial oxidative phosphorylation; thus, mitochondria play important roles in osteoclast fate determination [44]. Several lines of evidence support the idea that the process of osteoclast differentiation was accompanied by a significant increase in the number and volume of mitochondria, and a promotion of the expression levels of mitochondrial respiratory chain complex genes [45,46]. The RNA-sequencing analyses in this study hinted that METTL3 might regulate mitochondrial activity during inflammatory osteoclastogenesis, and this was further verified in the LPS-stimulated osteoclasts. Our results showed that a METTL3 deficiency suppressed the expression of mitochondrial energy metabolism transcription factor and respiratory chain complex genes. The ATP concentration and mitochondrial membrane potential also decreased in METTL3-silenced cells. These results validated that METTL3-depletion-triggered mitochondrial dysfunction during LPS-induced osteoclastogenesis.

To further clarify the potential mechanism by which METTL3 regulates the mitochondrial function of osteoclasts, we sorted the differentially expressed genes and discovered that *Nos2*, which encodes the iNOS protein to catalyze NO synthesis, was the most obviously upregulated gene. NO is a multifunctional biomolecule produced by nitric oxide synthase (NOS) that exerts dual regulatory effects on mitochondrial function. The basal concentration of NO synthesized by constitutive NOS (cNOS) under physiological conditions exerts a protective effect on mitochondrial homeostasis, while excessive generation of NO catalyzed by activated iNOS in pathological environments can damage mitochondria and lead to cell apoptosis [27,47,48]. The high concentration of NO catalyzed by iNOS has been shown to inhibit the function of the mitochondrial electron transport chain (ETC) and reduce ATP synthesis by competing with oxygen to bind cytochrome c oxidase [49]. The peroxynitrite anion (ONOO^−^) produced due to the interaction of NO with superoxide anion (O_2_^−^) is a strong oxidant that causes peroxidation of the mitochondrial membrane lipid layer and destroys mitochondrial integrity [50]. In the present study, the biological experiment results confirmed that METTL3 silencing promoted the expression of *Nos2* mRNA and iNOS protein, and increased the content of NO during LPS-induced osteoclastogenesis. To further determine the role of iNOS/NO signaling in mitochondrial dysfunction, the NOS inhibitor L-NAME was then utilized in METTL3-silenced cells. The results showed that the ATP content and mitochondrial membrane potential were rescued upon L-NAME treatment. Furthermore, L-NAME treatment could rescue the inhibited osteoclast formation and suppress apoptosis of osteoclasts. Together, these results indicated that METTL3 silencing modulated the mitochondrial function and osteoclast development via iNOS/NO signaling during LPS-induced osteoclastogenesis.

The methyltransferase complex with METTL3 as the core catalyzes the formation of m^6^A in target mRNAs, which modulates gene expression at the post-transcriptional level by affecting RNA processing [34]. Through querying the transcriptomic data from the m^6^A database, we discovered that a typical RRACH consensus motif was contained in *Nos2* mRNA. M^6^A modifications were found to be enriched in the 3′ UTR and 5′ UTR regions of *Nos2* transcripts, implying that *Nos2* may be regulated by m^6^A methylation. In the present study, due to the upregulated expression of *Nos2* upon METTL3 knockdown, we speculated that METTL3 might promote the degradation of *Nos2* mRNA. The mRNA stability assay verified that the half-life of *Nos2* mRNA was prolonged after METTL3 knockdown, indicating that METTL3 silencing increased *Nos2* expression by enhancing the stability of mRNA. m^6^A methylation performs biological functions with the participation of specific m^6^A-binding proteins, predominantly including YTH domain-containing proteins [51]. Previous studies have shown that YTHDF1 and YTHDF2 accelerate the translation efficiency and degradation of m^6^A-methylated mRNAs, respectively, while YTHDF3 assists YTHDF1/2 to affect the fate of mRNA [52,53,54]. Nevertheless, researchers have successively stated in 2020 that YTHDF1/2/3 proteins have similar intracellular localization and RNA binding preferences. The three m^6^A “readers” function together to accelerate the degradation of m^6^A-modified mRNAs [29]. Since then, a growing body of research has demonstrated that YTHDF1, analogous to YTHDF2, controls various biological processes by influencing mRNA degradation [25,55]. In this study, YTHDF1 knockdown increased the stability and expression of *Nos2* mRNA, which was consistent with the effect of METTL3 silencing. YTHDF2 knockdown, however, did not affect the *Nos2* mRNA expression or half-life. The results of RIP-qPCR further confirmed that the YTHDF1 protein could directly bind to *Nos2* mRNA and the binding was weakened after METTL3 silencing. The findings suggested that METTL3-mediated m^6^A might regulate *Nos2* mRNA degradation and expression via YTHDF1.

Based on the inhibitory effects of METTL3 silencing on inflammatory osteoclast differentiation and bone resorption in vitro, further experiments were performed to explore the potential role of METTL3 in pathological bone loss in vivo. We constructed an LPS-induced cranial osteolysis mouse model, as previously described, and locally injected lentivirus-carrying shRNAs [56,57]. As evaluated through Micro-CT scanning, the injection of lentivirus-carrying *Mettl3*-shRNA significantly suppressed LPS-induced osteolysis. Quantitative analyses of bone morphology showed that *Mettl3*-shRNA interference rescued the alteration of BV/TV, BMD, Tb. N, Tb. Th and Tb. Sp caused by LPS administration. The results of histological analyses using H&E and TRAP staining indicated that a *Mettl3*-shRNA injection reduced bone erosion and the number of osteoclasts. These in vivo findings were in accordance with the results of in vitro experiments, indicating that METTL3 could modulate osteoclast development and might be considered a therapeutic target for inflammatory osteolysis. Despite these promising effects of METTL3 on osteoclast activities, there are some limitations to our study. Given that bone homeostasis is maintained by a dynamic balance between osteoblasts and osteoclasts, the effect of METTL3 on osteoblast biology in inflammatory bone diseases requires further investigation.

**Figure 8:**
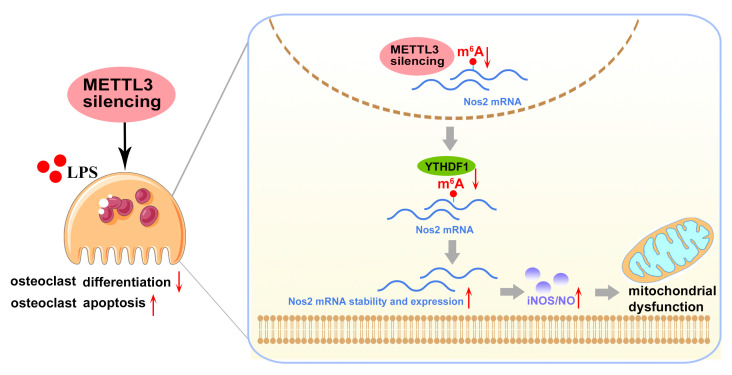
Mode pattern. METTL3 regulates osteoclast biological behaviors via iNOS/NO-mediated mitochondrial dysfunction in inflammatory conditions. METTL3 knockdown reduces YTHDF1-mediated *Nos2* mRNA degradation and promotes mRNA stability and expression of *Nos2*, which aggravates iNOS/NO-mediated mitochondrial dysfunction and, consequently, inhibits osteoclast differentiation and accelerates osteoclast apoptosis in inflammatory conditions. The red upward arrows mean upregulation; the red downward arrows mean downregulation.

In conclusion, our study demonstrated that lentivirus-mediated METTL3 silencing suppressed the inflammatory osteoclast differentiation and bone-resorbing capacity in vitro, as well as protecting against LPS-induced osteolysis in vivo. Mechanistically, METTL3 silencing enhanced the stability and expression of *Nos2* mRNA in a YTHDF1-dependent manner and aggravated iNOS/NO-mediated mitochondrial dysfunction, which, consequently, impaired osteoclastogenesis and promoted osteoclast apoptosis in the inflammatory environments (Figure 8). These findings uncovered the potential role of METTL3 in modulating LPS-induced osteoclastogenesis. Given the importance of osteoclast activity in the pathogenesis of inflammatory bone disorders, this study may provide a novel epigenetic perspective for future research on inflammatory osteolytic diseases. However, since the regulatory role of METTL3-mediated m^6^A is complicated and exhibits cell-type specificity, the precise function and underlying regulatory mechanisms of m^6^A in different cells and tissues during the development of inflammatory diseases need additional in-depth studies.

## 4. Materials and Methods

### 4.1. Cell Culture and Differentiation

Bone marrow cells were isolated from the femora and tibias of 5–8-week-old C57BL/6 mice (Animal Center of Sun Yat-sen University) and cultured in α-MEM supplemented with 10% FBS for 1 day. The suspended cells were collected and incubated with M-CSF (30 ng/mL) (Sino Biological, Beijing, China) for another 3 days to obtain bone marrow-derived macrophages (BMMs). RAW264.7 cells (murine macrophage cell line) acquired from the American Type Culture Collection (ATCC; Manassas, VA, USA) were cultured with α-MEM (Gibco, New York, NY, USA) containing 10% fetal bovine serum (FBS, Gibco, Carlsbad, CA, USA). Then, BMMs and RAW264.7 cells were employed as osteoclast precursor cells.

For LPS-induced osteoclastogenesis, BMMs were pretreated with 50 ng/mL RANKL (R&D Systems, Minneapolis, MN, USA) for 1.5 days. Then, LPS from *Escherichia coli* O55:B5 (Sigma-Aldrich, St. Louis, MO, USA) was used to stimulate BMMs for 4 days. BMMs were treated with M-CSF (30 ng/mL) throughout differentiation. RAW264.7 cells were pretreated with RANKL (50 ng/mL) for 1 day and further stimulated with LPS for another 2 days. BMMs and RAW264.7 cells not stimulated by RANKL and LPS were the negative control cells.

### 4.2. Cell Transfection

For constructing Mettl3-knockdown cells, a lentiviral vector GV248 carrying *Mettl3*-targeting sequence or nonspecific sequence (Table 1), along with pHelper 1.0 and pHelper 2.0 vectors (GENECHEM, Shanghai, China), were transfected into HEK293T cells. The packaged lentivirus was collected and used to infect BMMs and RAW264.7 cells that were then screened for stable clones using 6 ug/mL puromycin (Sigma-Aldrich, St. Louis, MO, USA).

Small interfering RNA (siRNA) was designed and synthesized to knock down Ythdf1 and Ythdf2 in RAW264.7 cells. Briefly, cells were transfected with siYthdf1, siYthdf2 or the nontarget siRNA (Invitrogen, Carlsbad, CA, USA) at a concentration of 50 nM using Lipofectamine™ RNAiMAX (Invitrogen, Carlsbad, CA, USA). Table 2 shows the sequence of the indicated siRNA. After 12 h of transfection, the culture medium was replaced and the cells were induced for osteoclast differentiation.

### 4.3. Cell Viability Assay

BMMs and RAW264.7 cells were seeded onto the 96-well plates and cultured in α-MEM supplemented with 10% FBS and different concentrations of LPS (0, 100, 200, 500, 1000 ng/mL) for 4 days. Cell viability was determined by adding Cell Counting Kit-8 (CCK8; Dojindo Laboratories, Kumamoto, Japan) reagent and recording the absorbance at 450 nm with a microplate reader (Bio Tek Instruments, Winooski, VT, USA).

### 4.4. Tartrate-Resistant Acid Phosphatase (TRAP) Staining

For TRAP staining, cells were fixed for 20 min in 4% paraformaldehyde and then stained, as recommended by the manufacturer (Sigma-Aldrich, St. Louis, MO, USA). Cells stained purple with three or more nuclei were considered mature osteoclasts.

### 4.5. Bone Resorption Assay

BMMs and RAW264.7 cells cultured on 24-well Osteo Assay Surface plates (Corning Incorporated Life Science, Corning, NY, USA) were induced to differentiate into osteoclasts. Sodium hypochlorite solution and ultrapure water were used to clean the plates. The resorption areas were imaged using an inverted microscope (Axio Observer 5, Zeiss, Jena, Germany). The pit formation area proportion was quantified using ImageJ v1.47 software (National Institute of Health, Bethesda, MD, USA).

### 4.6. Flow Cytometry Assay of Apoptosis

Cell apoptosis was detected using an Annexin V-APC/7AAD Apoptosis Kit (Elabscience, Wuhan, China). RAW264.7 cells were pretreated with RANKL and then stimulated with LPS for 3 days. Then, osteoclasts were collected and incubated with Annexin V-APC and 7AAD in the dark for 20 min following the manufacturer’s instructions. The apoptosis rate was measured using a CytoFLEX flow cytometer (Beckman Coulter, Brea, CA, USA) and analyzed with FlowJo vX0.7 software (FlowJo LLC, Ashland, OR, USA).

### 4.7. Real-Time Quantitative Polymerase Chain Reaction (qRT-PCR)

RNA was isolated using the RNAzol reagent, and cDNA was generated with PrimeScript™ RT master mix (Takara, Tokyo, Japan). The qRT-PCR analysis was performed using a Light Cycler 96 instrument (Roche, Basel, Switzerland). The housekeeping gene *β-Actin* was used as a reference to determine the relative mRNA expression of objective genes. All primers required for the experiment are listed in Table 3.

### 4.8. Quantitative Analysis of m^6^A Levels

Total RNA was extracted from cells and purified using RNAZol (MRC, Cincinnati, OH, USA). For m^6^A quantification, 200 ng of RNA was collected. The m^6^A levels were measured with an EpiQuik m^6^A RNA Methylation Quantification kit (Colorimetric) (EpiGentek, Farmingdale, NY, USA) according to the manufacturer’s instructions.

### 4.9. RNA Sequencing

Cells transfected with *Mettl3*-targeting shRNA and the negative control shRNA were differentiated into osteoclasts and then lysed in RNAzol to isolate RNA. A total of 1 μg total RNA for each sample was prepared for sequencing using the BGI-500SEQ sequencer provided by BGI Tech (Shenzhen, China). The transcript expression was quantified as fragments per kilobase per million reads (FPKM). Bioinformatic analyses, including Kyoto Encyclopedia of Genes and Genomes (KEGG) analysis, gene ontology (GO) analysis, and gene set enrichment analysis (GSEA) were performed using the BGI data analysis platform and the OmicStudio tools at https://www.omicstudio.cn/tool (accessed on 30 November 2020).

### 4.10. Western Blotting Analysis

The protein was obtained from cells using the RIPA lysis buffer (Beyotime, Haimen, China) and quantified using a BCA protein assay kit (Beyotime, Haimen, China). For Western blot analysis, 25 μg protein was separated using 8–12% sodium dodecyl sulfate-polyacrylamide gel electrophoresis (SDS-PAGE) and then transferred onto polyvinylidene fluoride (PVDF) membranes (Millipore, Billerica, MA, USA), which were blocked with 5% BSA for 1.5 h. Then, membranes were probed with primary antibodies overnight at 4 °C followed by incubation with secondary antibodies (1:2000; Cell Signaling Technology, Boston, MA, USA) for 1 h at room temperature. Primary antibodies included METTL3, YTHDF1, YTHDF2, BCL2, caspase-9, caspase-3 (1:1000; Proteintech, Chicago, IL, USA), BAX (1:1000; Affinity Biosciences, Cincinnati, OH, USA), NFATC1, c-FOS, iNOS, β-ACTIN (1:1000; Cell Signaling Technology, Boston, MA, USA), CTSK and TRAP (1:1000; Abcam, Cambridge, UK). The specific protein bands were visualized using enhanced chemiluminescence (Millipore, Billerica, MA, USA). The band densities were quantified with ImageJ software (National Institutes of Health, Bethesda, MD, USA).

### 4.11. Nitric Oxide Assay

The concentration of nitric oxide in the cell culture supernatant was assessed using Griess Reagent following the instructions for the Nitric Oxide Assay Kit (Beyotime, Haimen, China).

### 4.12. ATP Level Detection

For measuring intracellular ATP concentration, an ATP Assay Kit (Solarbio, Beijing, China) was used following the manufacturer’s recommended protocol.

### 4.13. Mito-Tracker Red CMXRos Staining

After osteoclastic induction using LPS, cells were incubated with 50 nM Mito-Tracker Red CMXRos (Beyotime, Haimen, China) for 20 min at 37 °C according to the manufacturer’s instructions. The nuclei were stained with DAPI (Beyotime, Haimen, China) for 5 min at room temperature. The images were obtained with a laser scanning confocal microscope (LSM980, Zeiss, Jena, Germany). The fluorescence intensity was analyzed using ImageJ software (National Institutes of Health, Bethesda, MD, USA) to reflect mitochondrial membrane potential.

### 4.14. RNA Stability Measurement

The mRNA transcription within cells was blocked through stimulation with 5 μg/mL transcription inhibitor actinomycin D (Sigma-Aldrich, St. Louis, MO, USA). RNA was obtained at 0, 3 and 6 h after actinomycin D treatment and then used for qRT-PCR analysis. The stability of mRNA was evaluated by analyzing relative mRNA expression at the relevant time points.

### 4.15. RNA-Binding Protein Immunoprecipitation (RIP)

A Magna RIP Kit (Merck Millipore, MA, USA) was used for RIP according to instructions. In brief, prepared cells were collected and lysed in the RIP lysis buffer after being washed with ice-cold PBS. Magnetic beads conjugated with YTHDF1 and IgG antibodies were prepared and used for incubating the cell lysates overnight at 4 °C. The immunoprecipitate was washed with cold RIP Wash Buffer and incubated with proteinase K for 30 min at 55 °C. The bound RNA was isolated using RNAzol reagent and analyzed using qRT-PCR.

### 4.16. Establishment and Analysis of LPS-Induced Osteolysis Murine Model

The construction of the LPS-induced osteolysis murine model in mouse calvaria was carried out as previously reported [30]. A total of 24 female C57/BL6 mice aged 6 weeks were randomly assigned to the following 4 groups (*n* = 6 each): Group 1, PBS; Group 2, LPS (25 mg/kg); Group 3, shCtrl (1 × 10^7^ TU) + LPS (25 mg/kg); Group 4, shMettl3 (1 × 10^7^ TU) + LPS (25 mg/kg). The regents were administered subcutaneously over the sagittal midline of the murine calvaria. LPS was injected every three days for 6 days in Groups 2, 3 and 4, while PBS was injected in Group 1. Lentivirus (1 × 10^7^ TU) carrying the negative control shRNA or *Mettl3* shRNA was injected 2 days before the first LPS treatment in Group 3 and Group 4. Mice were sacrificed after 6 days of LPS injection and their calvaria were separated and fixed with 4% paraformaldehyde for 2 days.

A Micro-CT scanner (μCT 50; SCANCO Medical AG, Basserdorf, Switzerland) was used for calvaria scanning and three-dimensional reconstruction. The scanning parameters were as follows: resolution, 10 μm; source voltage, 70 kV; current, 114 μA; aluminum filter, 0.5 mm; exposure time, 300 ms. After reconstruction, the square region of interest around the midline suture was chosen for quantitative analysis. The parameters analyzed for bone morphometry included the bone volume fraction (BV/TV), bone mineral density (BMD), trabecular number (Tb. N), trabecular thickness (Tb. Th) and trabecular separation (Tb. Sp). For histological analysis, the calvaria were embedded in paraffin after being decalcified in 10% EDTA for 3 weeks. Sectioned tissues were prepared for hematoxylin and eosin (H&E) and TRAP staining following the manufacturer’s protocols.

### 4.17. Statistical Analysis

Data from three independent replicated experiments were presented as the mean ± standard deviation (SD). GraphPad Prism 8 software (San Diego, CA, USA) was used for statistical analyses. The Shapiro–Wilk test was performed to check for normality and revealed that the data presented a normal distribution. Student’s *t*-test or one-way analysis of variance (ANOVA) was performed to evaluate statistical significance, considering *p* < 0.05 as significant.

## Figures and Tables

**Table 1:** Target sequences of shRNA.

shRNA	Target Sequences
shMettl3	GCACCCGCAAGATTGAGTTAT
shCtrl	TTCTCCGAACGTGTCACGT

**Table 2:** Target sequences of siRNA.

siRNA	Target Sequences (5′-3′)
siYthdf1	CCCGUAUCUCACUACCUAUTT AUAGGUAGUGAGAUACGGGTT
siYthdf2	CCAUGAUUGAUGGACAGUCAGCUUU AAAGCUGACUGUCCAUCAAUCAUGG
siCtrl	Stealth RNAiTM siRNA Negative Control LO GC (12935-200)

**Table 3:** Primer sequences for qRT-PCR.

Gene	Forward Primer (5′-3′)	Forward Primer (5′-3′)
*Mettl3*	CTTTCTACCCCATCTTGAGTG	CCAACCTTCCGTAGTGATAGTC
*Ctsk*	CACCCAGTGGGAGCTATGGAA	GCCTCCAGGTTATGGGCAGA
*Acp5*	ACCTTGGCAACGTCTCTGCAC	GTCCAGCATAAAGATGGCCACA
*Nfatc1*	CCCGTCACATTCTGGTCCAT	CAAGTAACCGTGTAGCTGCACAA
*c-fos*	CGGCATCATCTAGGCCCAG	TCTGCTGCATAGAAGGAACCG
*Pgc-1β*	CTTGGCTGCGCTTACGAAGA	GAAAGCTCGTCCACGTCAGAC
*Ndufb10*	GATTCTTGGGACAAGGATGTGT	CCTTCGTCAAGTAGGTGATGGG
*Sdha*	AATTTGCCATTTACCGATGGGA	AGCATCCAACACCATAGGTCC
*Uqcrc2*	AAAGTTGCCCCGAAGGTTAAA	GAGCATAGTTTTCCAGAGAAGCA
*Cox7a1*	ACAATGACCTCCCAGTACACT	CCAAGCAGTATAAGCAGTAGGC
*Nos2*	ACATCGACCCGTCCACAGTAT	CAGAGGGGTAGGCTTGTCTC
*Ythdf1*	ACAGTCCAATCCGAGTAACAGT	GGTAGTGAGATACGGGATGGGA
*Ythdf2*	AGGCGGGTTCTGGATCTACT	ACCCGGCCATGTTTCAGATT
*β-Actin*	CATACCCAAGAAGGAAGGCTGG	GCTATGTTGCTCTAGACTTCGAGC

## Data Availability

Data supporting reported results are available from the corresponding authors to all interested researchers.
